# Systemic administration of strontium ranelate to enhance the osseointegration of implants: systematic review of animal studies

**DOI:** 10.1186/s40729-018-0132-8

**Published:** 2018-07-17

**Authors:** Cassio Rocha Scardueli, Carolina Bizelli-Silveira, Rosemary Adriana C. Marcantonio, Elcio Marcantonio, Andreas Stavropoulos, Rubens Spin-Neto

**Affiliations:** 1 Department of Dentistry and Oral Health, Aarhus University, Aarhus, Denmark; 2 Department of Periodontology, São Paulo State University (Unesp), School of Dentistry, Araraquara, São Paulo Brazil; 3 Department of Periodontology, Faculty of Odontology, University of Malmö, Malmö, Sweden

**Keywords:** Strontium, Systemic use, Osseointegration, Bone remodeling

## Abstract

The literature states that Strontium (Sr) is able to simultaneously stimulate bone formation and suppress bone resorption. Recent animal studies suggest that the systemic administration of Sr, in the form of strontium ranelate (SRAN), would enhance the osseointegration of implants. The purpose of the present study was to undertake a systematic review on animal studies evaluating the systemic administration of Sr to enhance the osseointegration of titanium implants and the remodeling of bone grafts. The MEDLINE (PubMed) and Scopus bibliographic databases were searched from 1950 to October 2017 for reports on the use of systemic and non-radioactive Sr to enhance the osseointegration of titanium implants and the remodeling of bone grafts in animals. The search strategy was restricted to English language publications using the combined terms: “strontium” and “implant or graft or biomaterial or bone substitute”. Five studies were included, all related to the systemic administration of Sr in the form SRAN, and its effects on osseointegration of titanium implants. No studies on the use of SRAN-based therapy to enhance the remodeling of bone grafts were found. The studies differed notably with respect to the study population (healthy female rats, healthy male rats, and female rats with induced osteoporosis) and SRAN dose (ranging from 500 to 1000 mg/kg/day). Results were diverse, but a tendency suggesting positive influence of systemic SRAN administration on the osseointegration of titanium implants was observed. No major side-effects due to strontium administration were reported. Systemic Sr administration, in the form of SRAN, seems to enhance peri-implant bone quality and implant osseointegration in animals, however, at a moderate extent. Further studies, evaluating both the effects of this drug on implant osseointegration and the risk/benefit of its use, are needed to provide a rationale of this therapeutic approach.

## Introduction

Following the trauma induced to the bone tissue during dental implant installation, wound healing involves the fine-tuned coupling of bone resorption and formation [1, 2], which finally leads to the direct bone-to-implant contact, i.e., implant osseointegration [3]. The same biological mechanisms are involved in the wound healing (i.e., remodeling) of a bone defect filled with bone graft and/or bone substitute material [4]. In order to enhance wound healing and thereby achieve an optimized osseointegration and/or bone defect closure, systemic-, and local administration of drugs, including growth and differentiation factors, and/or implant and graft drug-based surface modifications have been employed with variable success [5, 6]. In the past years, the literature regarding the local interventions (mostly based on the modification of implant surfaces to improve the osseointegration process) became highly developed [6]. On the other hand, the additional effect of systemic therapies supplementing such local modification factors, although acknowledged, was not developed in a similar manner [5].

Examples of systemic administration of anabolic and anti-catabolic substances include estrogen [7], parathyroid hormone [8], and bisphosphonates [9, 10]. In particular, systemic administration of bisphosphonates, which are widely used for cancer and osteoporosis treatment, is based on the rationale that suppression of bone resorption—achieved by this type of drugs—results in denser peri-implant bone and thereby in larger amounts of bone-to-implant contact [11]. Indeed, a recent review of animal studies indicated that systemic administration of bisphosphonates enhances implant osseointegration, especially in animals with induced osteoporotic conditions [12]. Nevertheless, the increasing number of reports in recent years of bisphosphonate-related osteonecrosis of the jaws has raised alarm regarding the side-effects of bisphosphonate treatment [13] and has led to the search of alternatives to this group of drugs.

Another type of drug recently developed for osteoporosis treatment is strontium (Sr) ranelate (SRAN) [14–16]. This drug is a salt consisting of two atoms of stable strontium (Sr^2+^) and an organic acid (Ranelic acid), and it is usually administrated orally [17, 18]. Sr ions possess a high affinity to hydroxyapatite (HA) [19], and in contrast to bisphosphonates that decrease bone resorption, Sr exerts a dual action, i.e., it is able to simultaneously stimulate bone formation and suppress bone resorption [17, 20, 21]. This has been demonstrated in both animal- (by means of bone mineral content analysis [22], dual-energy X-ray absorptiometry [23], and histomorphometric assessment [24]), and clinical studies (by means of microtomography and histomorphometric assessment) [25]. Further, relatively recent reports from animal studies suggest that the systemic administration of Sr would enhance the osseointegration of implants.

Thus, the aim of this review was to undertake a systematic review of the literature on the available evidence—deriving from animal studies—on the systemic administration of non-radioactive Sr to enhance the osseointegration of titanium implants and/or the bone regeneration (i.e., remodeling) in association with bone grafting techniques.

## Material and methods

This review was executed in accordance with PRISMA (Preferred Reporting Items for Systematic Reviews and Meta-Analyses) [26], and the Cochrane Handbook for Systematic Reviews of Interventions [27].

### Criteria for considering studies for this review

Animal studies in which the methodology/results included parameters regarding the use of systemic administration of non-radioactive Sr to enhance the osseointegration of implants and/or the remodeling of bone (and bone substitute materials) grafts qualified for inclusion. Retrieval of information focused on (1) the used posology (dose and timing), (2) the type of treatment, (3) the assessed outcome, and (4) the side effects of the treatment. Studies which failed reporting one or more of these four topics still qualified for inclusion, but the missing information was acknowledged as non-declared.

### Search strategy for identification of studies

#### Electronic search

The MEDLINE (Medical Literature Analysis and Retrieval System Online, via PubMed) and Scopus databases were searched until October 2017 for studies evaluating the use of systemic non-radioactive Sr to enhance to enhance the osseointegration of titanium implants and the remodeling of bone grafts. The search strategy was restricted to English language publications using the combined terms: (strontium) and (implant or graft or biomaterial or bone substitute). Systematic reviews, reviews, and case reports were immediately excluded.

### Hand-searching

#### Unpublished data and hand-searching

Unpublished data were sought by searching a database listing unpublished studies (OpenGray-www.opengrey.eu). A manual search was additionally conducted based on the reference lists of the selected papers. Further, electronic databases of the following journals, which were considered important to this review, were separately manually searched: *Clinical Oral Implants Research*, *Bone*, *International Journal of Implant Dentistry*, *Osteoporosis International*, *Journal of Periodontology*, *Journal of Clinical Periodontology*, *Journal of Dentistry*, *Dentomaxillofacial Radiology*, and *Oral Surgery*, *Oral Medicine*, *Oral Pathology and Oral Radiology.* Further, the bibliographic references of the included studies were also sought for possible relevant studies.

Titles, abstracts, and full texts of the search results were independently screened in duplicate by three reviewers (CRS, CBS, and RSN). When there was a disagreement, the reviewers discussed the study and reached consensus.

### Study selection and data extraction

Three independent researchers (CRS, CBS, and RSN) conducted data extraction and validity assessment of the studies that met the inclusion criteria. Data was extracted focusing on the animal model, study groups, treatment start, duration of the treatment, period of examination, implant specification, evaluation methods, and results (outcomes), according to what was reported in each study (i.e., histomorphometry, biomechanical, microtomography, and serum analysis).

### Study outcomes

Any qualitative and/or quantitative bone-related parameters which could explain the effects of the systemic administration of non-radioactive Sr on bone tissue of the animals, determining if and how this substance interacts with the bone tissue and bone-tissue regeneration, were included as relevant outcomes.

### Quality assessment and risk of bias of included studies

Quality assessment of the studies was made according to SYRCLE’s risk of bias tool for animal studies [28]. All ten domains (sequence generation, baseline characteristics, allocation concealment, random housing, performance blinding, random outcome assessment, detection blinding, incomplete outcome data, selective outcome reporting, and other sources of bias) of SYRCLE’s tool were individually evaluated in terms of the risk of bias (no summary scores for the studies were included). Three reviewers (CRS, CBS, and RSN) independently assessed the studies for their quality. When there was a disagreement regarding the assessed data, a consensus meeting was carried out.

## Review

### Search results

The initial search for publications yielded 578 titles in MEDLINE (PubMed) database, and 152 in Scopus database. After duplicates were removed, there were a total of 553 titles to be screened. After initial screening, using the abstracts and key words, 37 publications remained (31 from PubMed and 6 from Scopus), that potentially met the inclusion criteria. Hand-searching did not reveal any additional publications. After full-text reading, publications which did not fulfill the inclusion criteria were excluded. From the selected full-texts, the main reason for exclusion was the methodology based on the local delivery of Sr, instead of its systemic use.

**Fig. 1:**
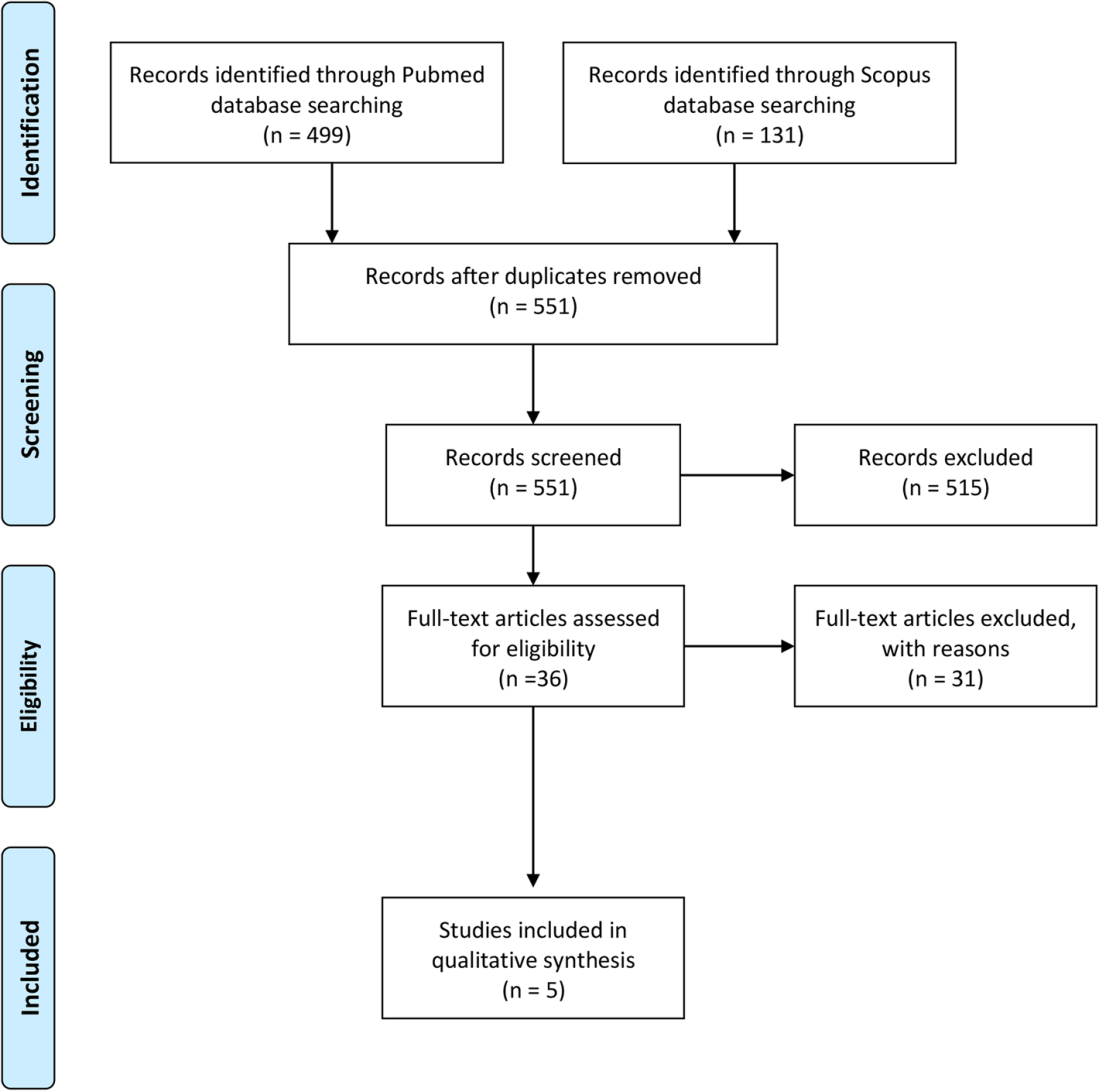
PRISMA flow diagram

Finally, five studies reporting on the impact of systemic administration of Sr on the osseointegration of titanium implants were identified as eligible to be included in this systematic review. No additional publications were found from the bibliographic references of the included studies. No information on the use of systemic administration of strontium to enhance the remodeling of bone or bone substitute materials following grafting procedures were found. The study selection procedure is presented in the PRISMA flowchart (Fig. 1).

### Study outcomes

Tables 1 and 2 show the information retrieved from the included studies. The studies differed notably with respect to the study population, Sr dose, time-point of medication start, duration of the treatment and period of evaluation, implant specification, and the methods.

**Table 1:** Included studies and sample characteristics. Animal model, study groups, treatment start point and its duration, period of evaluation, implants specification, and evaluation methods

Study		Sample characteristics				
	Animal model	Study groups	Treatment start	Duration of the treatment and period of examination	Implant specification	Evaluation methods
Maïmoun et al. 2010 [29]	Female rats	C: 0.5% carboxymethylcellulose aqueous solution (gavage, 5 days a week, *n* = 15) SRAN: 625 mg/kg of strontium ranelate, for 8 weeks following implantation (gavage, 5 days a week, *n* = 15)	nd	8 weeks	1.0 × 4.1 mm pure titanium, with sand-blasted, acid-etched surface, inserted in the tibial methaphysis, bilaterally	Microtomography, pull-out biomechanical test, and nanoindentation tests
Li et al. 2010 [32]	Female rats	C_S_: sham operation as a control group, and did not receive any drug treatments (*n* = 10) OVX: bilateral ovariectomy 12 weeks before implantation (*n* = 10) OVX + SRAN_L_: bilateral ovariectomy 12 weeks before implantation, plus special diet providing 500 mg/kg/day of strontium ranelate after implantation (*n* = 10) OVX + SRAN_H_: bilateral ovariectomy 12 weeks before implantation, plus special diet providing 1000 mg/kg/day of strontium ranelate after implantation (*n* = 10)	Started on the day of implantation	12 weeks	1.8 × 3.5 mm, sand-blasted and with sprayed HA-coating, inserted in the tibiae, unilaterally	Microtomography, push-out biomechanical test
Li et al. 2012 [33]	Female rats	OVX: bilateral ovariectomy, 12 weeks before implantation (*n* = 10) OVX + SRAN: bilateral ovariectomy 12 weeks before implantation, plus special diet providing 625 mg/kg/day of strontium ranelate after implantation (*n* = 10)	Started on the day of implantation	12 weeks	1.2 × 10.0 mm commercially pure titanium, machined and grit-blasted with aluminum oxide, inserted in the distal femur, bilaterally	Microtomography, histomorphometric (BA and BIC), serum analysis (OCN and TRAP), and push-out biomechanical test
Linderbäck et al. 2012 [30]	Male rats	C: normal diet (*n* = 20) BP_A_: 20 μg/kg/day subcutaneous alendronate, three times a week (*n* = 20) SRAN: special diet containing 800 mg/kg/day strontium ranelate (*n* = 20)	BP_A_ group started within 24 h after implantation SR group started on the day of implantation	4 and 8 weeks	1.6 × 2.5 mm stainless steel and polymethyl methacrylate (PMMA) screws, inserted in the tibial methaphysis, bilaterally	Microtomography, pull-out biomechanical test, histomorphometric analysis (BA), and bone mineral analyses (ash weight)
Chen et al. 2013 [31]	Female rats	C_S_: sham operation as a control group, and did not receive any drug treatments (*n* = 12) OVX: bilateral ovariectomy 4 weeks before implantation, and did not receive any drug treatments (*n* = 11) OVX+BP_A_: bilateral ovariectomy 4 weeks before implantation, alendronate 7 mg/kg/week orally (*n* = 10) OVX+SRAN*:* bilateral ovariectomy 4 weeks before implantation, strontium ranelate 500 mg/kg/day orally (*n* = 12) OVX+BP_Z_: bilateral ovariectomy 4 weeks before implantation, single dose of zoledronic acid 0.1 mg/kg intravenous (*n* = 11)	1 week after implantation	11 and 12 weeks	1.0 × 10.0 mm titanium plasma-sprayed with hydroxyapatite, inserted in the tibial methaphysis, bilaterally	Bone mineral density, histological analysis, and push-out biomechanical test

*C*_*S*_ sham operation as a control positive group, *C* control, *SRAN* strontium ranelate, *OVX* ovariectomy, *SRAN*_***L***_ strontium ranelate low dose, *SRAN*_H_ strontium ranelate high dose, *BP* bisphosphonate, *BP*_*A*_ alendronate, *BP*_*Z*_ zolendronate, *nd* non declared

**Table 2:** Included studies and the evaluation methods employed, together with major outcomes for each method

Study		Tests and overall results				
	Bone mineral density	Histomorphometric	Biomechanical	MicroCT	Nanoindentation	Serum analysis
Maïmoun et al. 2010 [29]	_	*_*	SRAN > C	SRAN > C (trabecular bone microarchitecture and BIC)	SRAN > C (elastic modulus, tissue hardness, and working energy)	_
Li et al. 2010 [32]	_	*_*	OVX + SR_H_=C_S_ > OVX + SRAN_L_ > OVX	OVX + SRAN_H_ > C_S_ > OVX + SRAN_L_ > OVX	_	_
Li et al. 2012 [33]	_	OVX + SRAN > OVX (BIC and BA)	OVX + SRAN > OVX	OVX + SRAN > OVX	_	OVX + SRAN > OVX (OCN) OVX + SRAN < OVX (TRAP)
Linderbäck et al. 2012 [30]	BP_A_ > SRAN=C (4 and 8 weeks)	BP_A_ > SRAN=C (4 and 8 weeks)	BP_A_ > SRAN=C (4 and 8 weeks)	BP_A_ > SRAN=C (around implant) BP_A_ > SRAN > C (growth plate)	_	_
Chen et al. 2013 [31]	BP_Z_ = C_S_ > BP_A_ = SRAN > OVX	BP_Z_ = C_S_ > BP_A_ = SRAN > OVX	BP_Z_ = C_S_ > BP_A_ = SRAN > OVX	_	_	_

*C*_*S*_ sham operation as a control positive group**,** *C* control, *SRAN* strontium ranelate, *OVX* ovariectomy, *SRAN*_L_ strontium ranelate low dose, *SR*_*H*_ strontium ranelate high dose, *BP* bisphosphonate, *BP*_*A*_ alendronate, *BP*_*Z*_ zolendronate, *BIC* bone contact implant, *BA* bone area, *OCN* osteocalcin, *TRAP* tartrate-resistant acid phosphatase

In one of the studies included in the present review, including healthy female rats, SRAN at a dose of 625 mg/kg/day induced 33.9% higher pull-out strength values, improved trabecular bone microarchitecture (bone volume/total volume, trabecular thickness, structure model index, and connectivity), bone biomechanical characteristics, and bone-to-implant contact, compared with a control (C) group [29].

In another study, including healthy male rats, SRAN at a dose of 800 mg/kg/day did not lead to higher implant pullout values, but showed increased bone volume fraction, trabecular number, and decreased trabecular separation compared with the control group, based on microtomographic and histomorphometric findings. Further, SRAN was not able to significantly enhance implant pull-out values when compared to the treatment with bisphosphonates (alendronate) of 4 and 8 weeks [30].

In three studies from the same research group, female rats with ovariectomy-induced (OVX) osteoporosis were included [31–33]. Although the same research group performed the studies, the results were based on diverse animal populations, as it can be inferred from the studies. Rats receiving a high dose of SRAN (SRAN_H_, 1000 mg/kg/day) showed an increased ratio between bone and total voxels in direct contact to the implant (1.9-fold) compared with a non-supplemented OVX group. Similar trends were observed regarding trabecular thickness (by 1.2-fold), bone volume/tissue volume (by 1.1-fold), trabecular number (by 90%), and connectivity density (by 85.1%), evaluated by microtomography. In fact, the OVX + SRAN_H_ group showed similar or even statistically significantly better values regarding the above-mentioned parameters when compared with a non-osteoporotic group, while the improvements induced with a smaller dose of SRAN (SRAN_L_, 500 mg/kg/day) did not reach statistical significance [32] compared with the control group. However, SRAN_L_ animals showed improved implant osseointegration (as assessed by microtomography and histomorphometric evaluation) compared to a non-supplemented OVX group [33], but did not enhance bone-quality-related parameters when compared with a group receiving bisphosphonates (zolendronated and alendronate) [31]. Animals receiving bisphosphonates, however, showed significantly increased bone mineral density, and bone-to-implant contact and implant push-out values, when compared with a non-supplemented OVX group [31].

### Quality assessment and risk of bias

According to SYRCLE’s risk of bias tool for animal studies [28], the studies were classified in relation to the risk of bias regarding their selection, performance, detection, attrition, and reporting characteristics. All studies successfully met the criterion of not reporting their outcomes in a selective manner, and all proposed aims and elaborated hypotheses were adequately addressed in the results and discussion sessions of the papers. On the other hand, most studies were unclear regarding relevant steps, e.g., the housing of the animals, allocation concealment of the animals, whether outcome assessment was blinded, and whether incomplete outcome data existed and were not reported. Another possible source of bias may be the lack of consistent reporting regarding selection and justification of the posology of Sr supplementation. All these parameters represent relevant biases regarding the findings of the selected studies. The overall scoring for the included studies is presented in Table 3.

**Table 3:** Assessment of risk of bias using SYRCLE’s tool

Risk of bias	Study				
	Maïmoun et al. 2010 [29]	Li et al. 2010 [32]	Li et al. 2012 [33]	Linderbäck et al. 2012 [30]	Chen et al. 2013 [31]
Selection					
Sequence generation	L	L	U	L	L
Baseline characteristics	L	L	L	L	L
Allocation concealment	U	L	U	U	U
Performance					
Random housing	U	U	U	U	U
Blinding	U	L	U	L	U
Detection					
Random outcome assessment	U	U	U	U	U
Blinding	U	U	L	U	U
Attrition					
Incomplete outcome data	U	U	U	U	U
Reporting					
Selective outcome reporting	L	L	L	L	L
Other sources of bias	H	H	H	H	H

*L* low risk of bias, *H* high risk of bias, *U* unclear

## Discussion

### Mechanism of action

Sr is a metabolic trace element closely related to calcium. Sr^2+^ ions are incorporated into bone by two main mechanisms: (a) a rapid uptake mechanism, dependent on osteoblast activity, whereby Sr^2+^ becomes absorbed via ion exchange processes with Ca^2+^ or binding to osteoid proteins, and (b) Sr^2+^ ions incorporate into the crystal lattice of the bone mineral phase. [34] When Sr^2+^ is present in higher levels than those required for normal cell physiology, it induces pharmacological effects on bone, through the activation of diverse cellular pathways [34].

Systemic use of non-radioactive Sr has showed promising results regarding the treatment of bone pathologies, such as osteoporosis and osteoarthritis [35–37], as it directly and positively interferes with bone mass, quality, and strength [38]. Further, in vitro studies have shown that Sr has the ability to increase bone formation [39, 40] while inhibiting osteoclast differentiation [41]. This dual action on bone remodeling, distinguishes Sr from the other traditional anti-osteoporotic agents, which either increase bone formation (intermittent parathyroid hormones) or inhibit bone resorption (bisphosphonates) [42]. Several mechanisms have been discussed for how systemic Sr-based therapy enhances bone formation [43–46]. Sr might activate the calcium-sensing (or another functionally different cation-sensing) receptor on osteoblasts, thereby leading to enhanced bone matrix production [46]. Another plausible mechanism is that Sr induces prostaglandin production and cyclooxygenase expression, thereby increasing osteoblastic differentiation [44]. Further, Sr might interact with fibroblast growth factor receptors, thereby increasing osteoblast synthetic activity [43]. At last, Sr also interacts with the mitogen-activated protein kinase (MAPK) signaling pathway, enhancing the differentiation of mesenchymal stem cells in osteogenic cells [45]. In regard with interfering with bone resorption, Sr enhances osteoprotegerin (OPG) expression in osteoblasts [40, 47, 48] thereby reducing osteoclastogenesis [42].

Based on this dual effect, studies have suggested the use of Sr as an adjunct therapy in situations where enhanced bone tissue formation is needed [24, 46, 49]. One could hypothesize that any event dependent on bone formation, such as the osseointegration of implants, could benefit of strontium supplementation, i.e., osseointegration is enhanced due to improved bone formation rate and bone quality surrounding the implants [1, 2].

### Sr and implant osseointegration

All studies included in this review tested the hypothesis that non-radioactive Sr supplementation would enhance implant osseointegration. Overall, the results of the included studies suggest that the systemic Sr administration enhances peri-implant bone quality and implant osseointegration, however to a moderate extent. Positive results regarding implant osseointegration and the quality of peri-implant bone tissue, as evaluated by various methods (microtomography, histomorphometry, biomechanical, nanoindentation, and serum analysis) were observed in studies with both osteoporotic and healthy animals [29, 30, 32, 33]. However, the effect of Sr was not as pronounced as that achieved with systemic administration of bisphosphonates [30, 31]. As mentioned, systemic administration of bisphosphonates is known to lead to denser peri-implant bone, larger amounts of bone-to-implant contact, and overall enhancement of osseointegration-related parameters [11, 12, 50]. However, the reports on bisphosphonate-related osteonecrosis affecting the jaws [13] have practically removed these drugs from the group of possible alternatives for enhancing osseointegration [51].

In the present review, Sr was always used in the form of SRAN. In contrast to what is reported for bisphosphonates, no reports of major side-effects due to the SRAN treatment were found in the literature. However, it is relevant to mention that the literature is limited regarding the risks and pitfalls associated to systemic SRAN therapy. In fact, one population study conducted in France showed that osteoporotic patients with a history of venous thromboembolism presented cardiovascular side effects related to SR (104 cases in 39 months) [52]. In the same study, another important side effect, cutaneous toxicity, was mentioned connected to the first few weeks of drug administration. In this context, Sr dosage may be an important issue. SRAN dose in the studies included in the present review varied immensely (from 500 to 1000 mg/kg/day). It is a known fact that the anabolic effects of SR on bone remodeling are dose-dependent [23, 24], and indeed the positive effects of SR herein were observed with the higher doses [32, 33]. Clinical studies show efficacy regarding reduction of fracture risk in postmenopausal women with osteoporosis with a SRAN dose of 2 g/day [53]. Thus, a corresponding effective dose in a human adult with 60 kg of body weight would be 30–60 g/day. Despite the fact that high serum levels of strontium are required in rats in order to generate significant anabolic bone response [54], one could consider that if a dose 15 and 30 times higher than the clinical dose would be needed to have an effect on osseointegration, this could lead to toxicity in humans [55–57]. Clearly, this issue deserves further investigation.

The variability in the results of the studies included in this review, may not only relate to SRAN dose, but may somehow relate to differences in the time-period after ovariectomy, before the animals were included in the study, which differed greatly among studies (from 4 to 12 weeks). Although it is already defined in the literature that initial osteoporosis features appear already at 4 weeks after ovariectomy [58], in not a single study included in this review, were the osteoporotic conditions after ovariectomy confirmed by a specific test [31–33]. Thus, comparison of animals 4 and 12 weeks after ovariectomy in terms of bone architecture characteristics may not be considered optimal, since they represent diverse stages of osteoporotic state [58]. In the same context, in some studies, SRAN treatment started the same day of implant installation [30–33], while in other studies, treatment started 7 days after implantation [31]; SRAN treatment duration also varied much from among studies (from 4 to 12 weeks). Considering the fact that implant osseointegration in rats is completed within maximum 8 weeks after installation [59] and the fact that it is not yet known for how long should SRAN be used before it exerts a measureable effect on bone architecture, differences in the time-point of SRAN treatment start and its duration may have contributed to the variability in the results. Finally, differences in the evaluation methods used in the various studies, ranging from the gold-standard for osseointegration assessment, i.e., histomorphometry, to diverse biomechanical tests, and microtomography that has the inherent drawback of metal artifacts hampering osseointegration evaluation [60], may have also contributed to the observed variability in the results.

### Quality assessment and risk of bias

According to SYRCLE’s tool for assessing risk of bias [28], most studies were unclear regarding relevant steps in the selection, performance, detection, and attrition characteristics. On the other hand, there was a low risk for bias related to outcome reporting, so that the conclusions were often and straight-forward related towards the listed aims. Finally, a high risk for other biases, mostly related to inconsistency when defining and reporting the SRAN posology was seen in all included studies, something highly relevant, since it could directly reflect on the results and interfere in the reproducibility of the studies.

## Conclusions

Based on the few studies included in this systematic review, it is possible to state that the systemic administration of Sr, in the form of SRAN, seems to enhance peri-implant bone quality and implant osseointegration, however, to a moderate extent. Further studies should focus on standardization of the study designs to properly assess the effects of Sr, including parameters such SRAN dose, administration start point, and duration of administration, further allowing the assessment of potential risks/benefits of SRAN use.

## Abbreviations

Medline: Medical Literature Analysis and Retrieval System Online
OVX: Ovariectomy
Sr: Strontium
SRAN: Strontium ranelate
SYRCLE: Systematic Review Centre for Laboratory animal Experimentation
